# Filling Proximal Cavities in Bicuspids and Molars

**Published:** 1879-01

**Authors:** J. A. W. Davis

**Affiliations:** GALESBURG.


### ARTICLE IV.

### *Filling Proximal Cavities in Bicuspids and Molars.*

BY J. A. W. DAVIS, GALESBURG.

Read before the Illinois State Dental Society.

Experience and observation have taught me that proximal cavities in bicuspids and molars are the most difficult cavities that we have to fill, in order to make them successful in saving the teeth.

I believe that I can safely assert that there are more failures in the fillings in these teeth by good operators, than any other class of fillings in the mouth.

The great difficulty of thoroughly filling these cavities seems to be universally acknowledged, and if I can only arouse thought and discussion that will lead to more thoroughness, and to greater success in the insertion of these fillings, I shall be satisfied. We have more large and difficult fillings to insert in this class of cavities than in any other location in the mouth, (and it is the large cavities in these teeth that I wish to call particular attention to in this paper,) and the nervous exhaustion that is inseparable from the faithful performance of our duty in these fillings, is, I presume, perfectly familiar to all. I have often deeply sympathized with patients in these long and tedious operations, and have long endeavored to make them as short as consistent with thoroughness, both for the benefit of the patient and myself.

The causes of so many failures in the fillings of proximal cavities in bicuspids and molars are the following: 1st. A defective preparation of the cavity, especially the cervical margin. 2d. Injury to the margin in inserting the filling, by over malleting. 3d. A want of thorough adaptation of the filling to the margins of the cavity. 4th. Improperly finishing these fillings.

In the preparation and filling of these cavities, is it possible for us to obviate these causes of our failures? It may be impossible to do so in every case, but I am inclined to the opinion that we can do so in a far greater number of cases than we have hitherto been doing, if we will only adopt better methods and earnestly endeavor to be more thorough in every detail of these operations.

These cavities should be opened from the crown surface with a fissure-bur, if there is not an opening there already. Should there be no opening on the crown surface, a thin file may be passed between the teeth, and from this separation we may rapidly cut and open the cavity by cutting down the enamel until the interior parts are entirely brought into view.

We should invariably apply the rubber dam in all cases, before preparation of the cavity is completed, so as to exclude all moisture, thereby enabling us to see that we get the margins all perfectly smooth.

I contend that no operator can prepare this class of cavities as perfectly without the dam as he can with it. Having the rubber dam securely adjusted so as to exclude all moisture, we proceed to remove all disintegrated dentine in the cavity. This being accomplished, the cervical margin should be cut away until a sound, solid, and a secure border is obtained, which should then be made as smooth as possible; an imperfect cervical margin being a very prolific cause of failure in these fillings.

I feel like urging extreme care in their preparation. We should not stop after a hasty and careless preparation and console ourselves with the delusive hope that it is all right, but we should in every case never stop short of an ever living consciousness that knows that this margin is prepared just as it should be, to avoid all danger from the recurrence of decay.

The buccal and palatal margins should next be cut away until a firm edge is secured, after which it should be thoroughly polished to facilitate the adaptation of the filling.

The cavity should now be so shaped as to permanently retain the filling. With suitable sized fissure-burrs and the engine we can very readily cut a groove or dovetail on each side of the cavity, if the tooth will admit of it; in some cases, where the dentine is very sensitive, I make two retaining pits on each side of the cavity, and follow up the fissure on the masticating surface, and make a retaining pit there unless both sides of the tooth are decayed, and if they are, I bridge across the crown surface, in nearly all cases of bicuspids, also in some cases with the molars, so as to make the filling perfectly secure from removal by mastication or any ordinary force that may be brought to bear upon it. There will very frequently be found in the cavities a deep undercut or overhanging projection of enamel at either the lingual or buccal corner, and sometimes at both; this should always be cut away, as it is almost impossible to so fill them as to avoid future fracture, and thus injury to the integrity of the entire filling.

Having the cavity properly shaped, and the margins all perfectly smooth and ready for the introduction of the filling, I now wish to call your attention to an appliance to aid in the filling of these cavities, that I have found to be very efficient in securing successful results, and one which greatly lessens the time and weariness both to myself and to the patient. I refer to the matrices introduced by Dr. Louis Jack, of Philadelphia, for filling proximal cavities in bicuspids and molars.

About a dozen pairs of these matrices are requisite to meet the varying cases that occur in practice; however, occasionally it will be necessary to have one of unusual size and form to meet special cases; when the space is very large, or when from fracture of the outer plate of enamel, a steel one will not remain in position. For this purpose one may be made of boxwood that will answer every purpose. Having, then, about a dozen pairs of these matrices, we select one of suitable size and shape, and with the pliers take it up and push it up between the teeth which are to be filled, until it presses pretty firmly upon the rubber dam and gum at the cervical margin, and impinges tightly between the teeth. It should now be wedged firmly against the tooth to be filled, with wedges of boxwood or other hard wood; these are to prevent it from moving while packing the filling. The matrix being properly adjusted to its place, the cavity is now ready for the reception of the gold.

It will be readily perceived that we have transformed this cavity from a compound proximal to a simple one by the use of this little matrix.

For commencing these fillings I generally use Williams' cylinders slightly annealed. If I do not start the filling from retaining pits, the cylinders used should just be large enough to maintain their places firmly when condensed. When I start from retaining pits I use No. $\frac{1}{2}$ and No. 1; after the retaining pits are perfectly filled, I take a cylinder long enough to reach from one retaining pit to the other. Great care should be taken to thoroughly adapt these first pieces of gold to the cervical and other margins, either with the mallet or hand pressure.

After we have the cervical margin well covered, the gold may then be annealed more and more as the filling progresses, until the cavity is pretty well filled, when No. 20 or 30 rolled gold should be used to finish the filling. Extreme care should be taken to consolidate the gold at all points as the filling progresses, paying particular attention to its solidity against the matrix where it comes in contact with the margins of the cavity. The filling being now properly introduced and condensed thoroughly, it only remains for us to remove the matrix and finish up the filling. It will be found if the selection and adjustment of the matrix has been correct, that very little filing and cutting down of the plug will be required, and if the packing has been carefully performed, that the gold will be solidly condensed at every part of the filling.

The finishing of the filling does not differ from the usual method pursued. I usually open still farther the lingual space, which is easily accomplished with suitable files and corundum disks. The peculiar form of the depression in the matrix produces a space which may be increased at pleasure. In many cases where the tendency to caries is very great, we should cut quite freely from the inner plates of enamel, doing this after both the adjoining fillings are put in, cutting down both gold and enamel together, allowing the fillings to touch only at the prominent outer parts. The result is then, an imitation of the exceedingly oval bicuspid; the immunity of which from decay all must have observed. Having thus far finished the filling with files and disks, I next take strips of emory cloth cut to the proper width, and with these polish the proximal margins, first using Nos. 1 and ½, and finally finishing with crocus, which gives a fine finish and a beautiful polish. I would urge care in finishing the cervical margins, never stopping our efforts at this point until we positively know that every part of the margin is perfectly smooth, with no projecting gold to induce a recurrence of decay, but on the other hand, we should continue to cut and polish at this point until the margin is perfection itself.

Then, if this margin was thoroughly prepared before the filling was introduced, and if the filling was perfectly adapted to it without injuring it by over malleting, and finally, if the cervical margins are perfectly dressed and polished, I think we have obviated the causes of failure in these fillings to a great extent.

I trust that those who have not tried this method of filling proximal cavities in bicuspids and molars, will do so at their earliest convenience. Simplicity of methods and greater success in the performance of our work are the two leading wants of our profession to-day. Notwithstanding the number of faithful operators is increasing, and the standard of operations is rising, the manner in which fillings are made by two-thirds of the dentists of this country is a reproach to us as a profession, and a disgrace to the age in which we live.

For the better serving of our patients, I have a hope that step by step the methods of filling may be made easier, thus within the capacity of a greater number, and more sure, until at length all may be able to reach very near perfection in this very difficult operation.—*Transactions.*

